# The efficacy and safety of Anyu Peibo Capsule in the treatment of patients with major depressive disorder in China: study protocol for a randomized placebo-controlled trial

**DOI:** 10.1186/s13063-021-05550-9

**Published:** 2021-09-03

**Authors:** Jingjing Huang, Yimin Yu, Yi Jiang, Wu Chen, Yan Li, Yifeng Shen, Qingshan Zheng, Huafang Li

**Affiliations:** 1 Shanghai Mental Health Center, Shanghai Jiao Tong University School of Medicine, 600 Wan Ping Nan Road, Shanghai, 200030 People’s Republic of China; 2 Su Zhou YiHua Biotechnology Co. Ltd., Suzhou, People’s Republic of China; 3 Shanghai University of Traditional Chinese Medicine, Shanghai, People’s Republic of China

**Keywords:** Depression, Anyu Peibo Capsule, Randomized controlled trials

## Abstract

**Background:**

Major depressive disorder is the second leading cause of years lost to disability worldwide. Anyu Peibo Capsule has been shown to be effective and safe in phase II trials.

**Methods:**

This clinical study is a multi-center, randomized, double-blinded, placebo-controlled, parallel-group, phase III trial of Anyu Peibo Capsule in China. The aim is to test whether the administration of Anyu Peibo Capsule compared to placebo improves clinical outcomes in adults (aged 18 to 65 years) with MDD. Patients will receive an 8-week treatment of Anyu Peibo Capsule 1.6 g per day or placebo. The primary outcome will be the change from baseline in the total score for the Montgomery-Asberg Depression Rating Scale at the end of the 8-week treatment.

**Discussion:**

The trial aims to provide pivotal evidence for the efficacy and safety of Anyu Peibo Capsule in patients with major depressive disorder.

**Trial registration:**

ClinicalTrials.govNCT04210973. Registered on December 26, 2019

## Background

Major depressive disorder (MDD) is a recurrent, disabling, and serious psychological disorder and the second leading cause of years lost to disability worldwide (World Health Organization [1]). Over the past two decades, China has experienced rapid changes which have had a huge impact on the lifestyles and mental health of individuals. Current epidemiological investigations have shown that the lifetime prevalence of MDD in China has reached 3.4% [2], meaning that there are about 50 million people with MDD requiring appropriate treatment.

Antidepressants play a prominent role in the treatment of MDD [3]. The Chinese guidelines for depressive disorders recommend antidepressants as a first-line treatment for MDD [4]. However, about 30% of patients fail to achieve remission despite treatment with multiple antidepressants [5]. Treatment continuity is one of the main challenges in treating patients with MDD [6]. A national survey in China has shown that the top reason (36.1%) for why patients with MDD discontinue their medication is *concern about long-term side effects* [7]. There is a need to develop novel treatments for the relief of the symptoms of depression and to increase patients’ tolerance for long-term treatment.

Anyu Peibo Capsule is a new antidepressant, extracted from *Piper laetispicum* C. DC. (Piperaceae), a climbing glabrous plant growing in the south of China which has been used for invigorating the circulation and reducing detumescence and stasis, as well as having analgesic properties [8–11]. The pharmacodynamics of Anyu Peibo Capsule have been assessed in healthy volunteers. A favorable safety profile, a trend that the curative effect was higher than that of placebo, and remission rate in MDD have been demonstrated for Anyu Peibo Capsule in randomized, blinded, and placebo-controlled phase IIa and IIb trials.

The ongoing clinical study is a Chinese multi-center, randomized, double-blinded, placebo-controlled, parallel-group, phase III trial of Anyu Peibo Capsule administration in adults (aged 18 to 65 years) with MDD (AYPB-MDD-III). Based on the results of a phase II study, this trial is designed to detect clinically relevant differences in clinical outcomes at the end of the 8-week treatment as the primary endpoint.

## Methods

### Trial design

AYPB-MDD-III is a multi-center, randomized, double-blinded, placebo-controlled, parallel-group phase III clinical trial of Auyu Peibo Capsule to treat adults with MDD. This clinical trial is sponsored by Su Zhou YiHua Biotechnology Co. Ltd. and conducted at Shanghai Mental Health Center and 13 other sites in China (Appendix 1).

### Study objectives

The primary objective is to investigate the efficacy and safety of Anyu Peibo Capsule in comparison with placebo in adults with MDD. An exploratory objective is to explore potential predictive biomarkers for efficacy.

### Participants and eligibility

Patients diagnosed with MDD at the time of screening will be enrolled across 14 sites in China. The trial period will be from July 2019 to December 2020. The inclusion, exclusion, and withdrawal criteria in AYPB-MDD-III (Table 1) have been chosen to exclude patients at risk of suicide.

**Table 1:** Inclusion criteria, exclusion criteria, and withdrawal criteria

Inclusion criteria	Exclusion criteria	Withdraw criteria
1. Adult (18~65 years old), outpatients or inpatients, male or female. 2. Patients with a primary diagnosis of major depressive disorder (MDD) based on the criteria of DSM-5, single episode or recurrent episode. 3. The total score of MADRS is ≥ 26 in both screening visit and baseline visit. 4. The first item of MADRS is ≥ 3 in both screening visit and baseline visit. 5. CGI-S is ≥ 4 in both screening visit and baseline visit. 6. The subject understands and consents to takes part in this clinical trial. The subjects should sign an informed consent form.	1. The subject has a current psychiatric diagnosis other than depression according to DSM-5, including schizophrenia spectrum disorders, bipolar and related disorders, anxiety disorders, obsessive-compulsive and related disorders, physical symptoms and related disorders, and other mental disorders. 2. The subject has a suicide attempt within the recent 1 year, has a currently significant risk of suicide, or has a score > 3 on item 10 (suicidal ideation) of MADRS. 3. The subject has a current depressive episode due to somatic general disease or a neurological disease, such as hypothyroidism. 4. When the MADRS total score of baseline visit compares with the screening visit, the decreasing rate is ≥ 25%. 5. Any unstable cardiovascular, hepatic, renal, blood, endocrine, or other medical diseases. 6. Any neurological disease (such as Parkinson’s disease, cerebrovascular accident, and epilepsy) or cerebral injury (traumatic or disease-related). Had a history or a high-risk related disease or medication of seizure disorder, except infantile febrile convulsion. 7. Known hypersensitivity to *Piper laetispicum* C. DC. or at least to two kinds of drugs. 8. Within 6 months before screening, there were addictions of alcohol and other substances (except nicotine). 9. The subject could not take medication or has a disease affecting drug absorption, distribution, metabolism, and excretion. 10. Clinically significant abnormal laboratory values (e.g., ALT or AST value above 2 times of clinical top limit; Cr value above normal top limit; thyroid gland function index (≥ 2 items in 5 items) above 1.2 times or below 0.8 times of the normal range, or investigator diagnosed with hypothyroidism or hyperthyroidism). 11. Clinically significant electrocardiographic (ECG) abnormalities in screening visit, such as QTc ≥ 450 ms in males or ≥ 470 ms in females. 12. The subject who used at least two different antidepressants with the recommended dose and adequate duration (maximum dosage by at least 4 weeks according to label) treatment still had no response. 13. The subject uses antidepressant drugs normally before 2 weeks of screening and stops using psychotropic drugs before randomization less than 5 half-life period (monoamine oxidase inhibitor: at least 2 weeks; fluoxetine: at least 1 month). 14. The subject received modified ECT, trans-cranial magnetic stimulation (TMS), vagus nerve stimulation (VNS), or systematic psychotherapy within 3 months. The subject received systematic light therapy, laser therapy and acupuncture, other traditional Chinese medicine, or systemic biofeedback therapy within 2 weeks. 15. Women who were pregnant, breastfeeding, or serum-HCG (+) on screening, or planning to become pregnant within 3 months after kick-off of the clinical trial. 16. Education level is below junior high school. 17. The subject has participated in a drug clinical trial within 1 month before screening. 18. The investigator thinks the subject is unsuitable to enroll in this clinical trial.	1. Occurrence of intolerable adverse events or serious adverse events. 2. Obvious protocol violation will impact the efficacy and safety assessment. 3. The subjects had symptoms of self-injury, suicide, manic episode, and with psychopathical feature. 4. The efficacy is not good. 5. The investigator determines to withdraw. 6. The subject will not continue the clinical trial or withdraw his/her informed consent. 7. Loss of follow-up. 8. Other circumstances in which the subject withdraw from the trial (such as changes in residence, which made it impossible to continue medication and follow-up).

Any of the following medications and treatments will be prohibited:

1. Other psychoactive drugs (except drugs permitted in the protocol), including antipsychotics, antidepressants, mood stabilizers, anti-anxiety drugs, and nootropics
2. Traditional Chinese medicines (including Chinese patent medicines and traditional Chinese medicine decoctions) which may have the effect of relieving depression
3. Modified electroconvulsive therapy, transcranial magnetic stimulation, vagus nerve stimulation, light therapy, laser therapy, acupuncture, other traditional Chinese medicine treatments, biofeedback therapy, and other treatment methods
4. Systematic psychotherapy, such as psychoanalysis and cognitive behavior therapy
5. Other medications and treatments that may significantly affect the efficacy and safety of antidepressants

Some combination therapies will be permitted:

1. For serious insomnia, zolpidem, zopiclone, S-Zopiclone, and zaleplon can be taken before sleep, as long as the dose does not exceed the upper limit specified in drug labels, and the cumulative dosing during the trial duration does not exceed 2 weeks.
2. Medications used to treat physical diseases with efforts made to ensure the type and dosage do not change during the trial.

The following are the termination criteria of the trial:

1. There is a major design error in the protocol which makes it difficult to evaluate the drug effect.
2. During the implementation of the trial, a major deviation occurs or an unexpected, serious risk to the patients is found.
3. The ethics committee or sponsor decides to terminate the clinical trial.

### Justification for no concomitant therapy

This trial is designed according to *Major Depressive Disorder: Developing Drugs for Treatment Guidance for Industry (FDA 2018)* [12] to develop a new drug. Following this guidance, randomized, double-blind, placebo-controlled, parallel designs are recommended as the current standard for short-term efficacy trials in MDD. When this trial was to be designed as no concomitant therapy, actions for risk control and management were included in the first consideration. Therefore, we will exclude those who are at risk of suicide and ensure appropriate and sufficient informed consent process. What is more, when the patients’ condition declines due to no treatment, the condition will be assessed to decide whether to unblind or not, the patient could withdraw at any moment, and then standard treatment will be provided.

### Randomization

Patients enrolled in the AYPB-MDD-III trial are allocated to treatment with Auyu Peibo Capsule or placebo in a 1:1 ratio via the clinical trial electronic central stochastic system (DAS) for interactive web response system (IWRS).

### Trial interventions

Auyu Peibo is a capsule containing 20 mg of extracts (main ingredient: amide alkaloids); the taste- and visual-masked placebo is an identically shaped capsule containing no effective materials. Four tablets of the study drug or placebo will be administered orally, with water, 30 min after breakfast and supper, twice per day for 8 weeks. The patient’s adherence is monitored and assessed by the percentage of the number of practical drugs to the number of prescribed drugs.

**Fig. 1:**
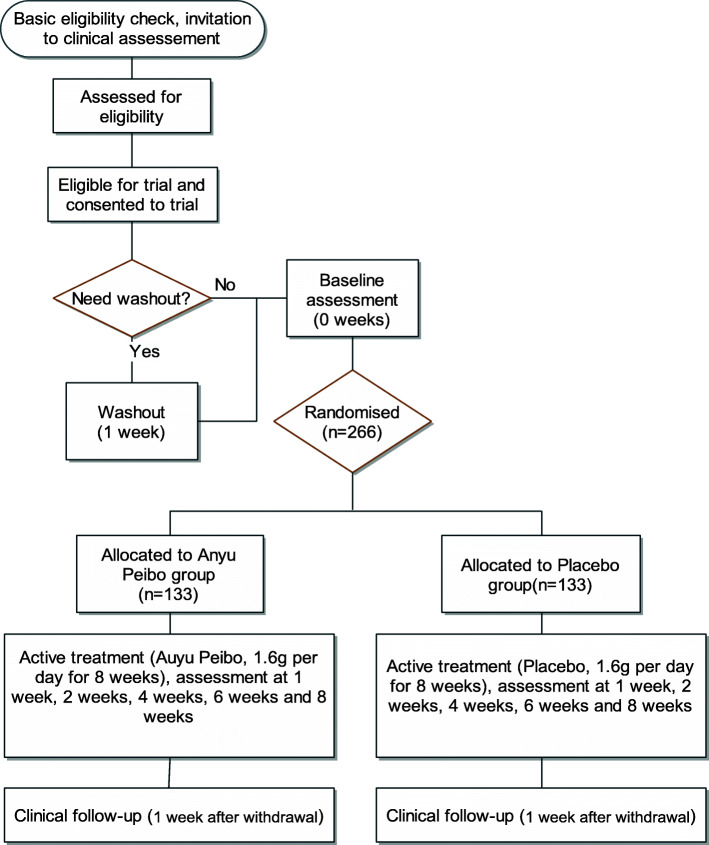
Participant flow through the AYPB-MDD-III

**Fig. 2:**
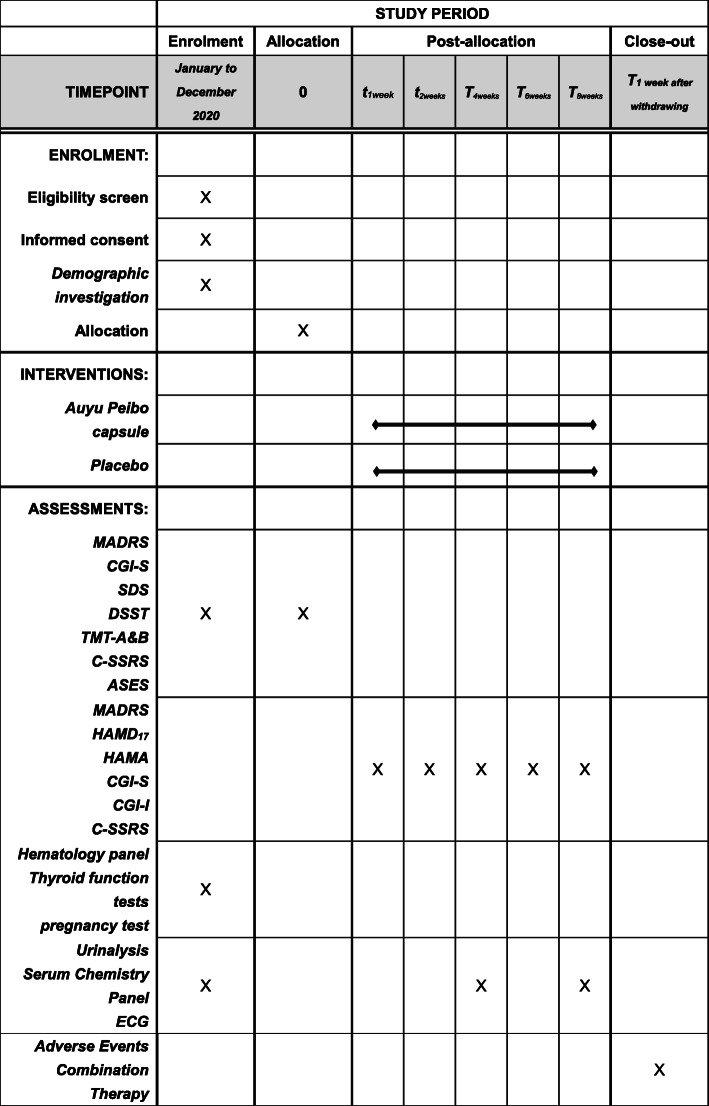
SPRIT scheme of study procedures

The patient flow is displayed in Fig. 1 according to the Consolidated Standards of Reporting Trials (CONSORT) [13] diagram, and the SPRIT [14] scheme of study procedures is shown in Fig. 2.

### Blinding

AYPB-MDD-III is double-blinded. Patients, care providers, investigators, and outcome assessors will not know the treatment allocation. The investigated drug is prepared by the central pharmacy and will be provided as a ready-to-use drug or placebo, labeled with a random number and patient number. The placebo is taste- and visual-masked. Emergency unblinding can be conducted via DAS for IWRS. Unblinding under emergency will be permissible due to a serious adverse event, or for the purpose of notification to the Data and Safety Monitoring Board (DSMB).

### Recruitment

Patients will be recruited from 14 sites (psychiatric hospitals) in China, where research psychiatrists and clinical staff from each site will invite potential patients to participate in the study.

### Data management and quality assurance

Trained research psychiatrists from each site will record the collected data on a source document and a web-based eCRF (DAS for EDC V6.0, in Chinese). Verification and cross-checking of the eCRF will be conducted by the study monitor, who will be provided by the site clinical research organization as well as the sponsor. Omissions, errors, and values requiring further clarification will be reviewed. Corrections should be made only by authorized personnel and be documented in an audit trail.

### Strategies to improve adherence to interventions

Reminder texts will be sent to patients before each study visit, and fees for public transportation will be reimbursed for attendance.

### Provisions for post-trial care

There will be no special post-trial care, and patients will continue their usual health care when the trial has been finished.

### Data collection methods

#### Study instruments

##### Chinese version of the Montgomery-Asberg Depression Rating Scale (MADRS)

The Chinese version of the MADRS [15, 16] has been translated from the original English version. It consists of 10 items rated on a 0–6 continuum (0 = no abnormality, 6 = severe) to assess the core symptoms of depression. Inter-rater reliability on the Chinese MADRS with different pairs of raters has been reported to be 0.954; reliability, validity, and sensitivity have been demonstrated to be good [17].

##### 17-item Hamilton Depression Scale (HAMD-17) Chinese version

The Chinese version of the HAMD-17 has been translated from the original English version. It consists of 17 items that are rated to assess the symptoms of depression. The Chinese HAMD-17 Cronbach’s alpha coefficients are calculated to be over 0.70, and internal consistency and validity have been demonstrated to be good [18].

##### Chinese version of the Sheehan Disability Scale (SDS)

The Chinese SDS measures impairment in work/school, social life, and family life responsibilities. The Chinese version of the SDS has been reported to have good validity and reliability [19].

### Primary outcome

The primary outcome is the change from baseline in the MADRS score following the 8-week treatment period.

### Secondary outcomes

The following secondary efficacy endpoints will be evaluated:

1. Clinical remission rate as assessed by the total MADRS score at the end of the study. Remission = at the end of the study, total MADRS score ≤ 10.
2. Clinical remission rate as assessed by the total HAMD-17 score at the end of the study. Remission = at the end of the study, total HAMD-17 score ≤ 7.
3. Clinical response rate according to the total HAMD-17 score at the end of the study. Response = at the end of the study, decreased rate (from baseline) for total MADRS score or total HAMD-17 score ≥ 50%.
4. Change in total MADRS score over time.
5. Change in total HAMD-17 score from baseline.
6. Change in Hamilton Anxiety Scale (HAMA) [20] total score from baseline.
7. Change in Clinical Global Impression-Severity of Illness (CGI-S) [21] score from baseline.
8. Improvement in Clinical Global Impression (CGI-I) [22] score at different visits.
9. Change in Discriminative Scale Space Tracker (DSST) [23] total score from baseline.
10. Change in Trail Making Test (TMT) A and B [24] total scores from baseline.
11. Change in the SDS [25, 26] total score from baseline.
12. Proportion of patients withdrawing due to poor efficacy. The investigators will assess patient efficacy according to his/her clinical status using rating scales, including MADRS, HAMD-17, HAMA, and CGI (already listed as outcomes).
13. Proportion of patients using combined medication to treat insomnia.

### Safety evaluation

Every patient enrolled in the trial will be assessed for the occurrence of adverse events (AEs). The incidence rate of AEs will be measured as the main safety outcome. The following measures will be used to evaluate the safety of the study drug:

1. Breath rate per minute, pulse rate per minute, heartbeat rate per minute, diastolic and systolic blood pressure, and sitting position (mmHg)
2. Electrocardiogram (ECG) and the number of patients with an abnormal 12-lead ECG report
3. Assessment of the Columbia-Suicide Severity Rating Scale
4. Assessment of the Arizona Sexual Experience Scale
5. Number of patients with AEs resulting in early withdrawal
6. Number of patients with serious adverse events (SAEs) resulting in early withdrawal
7. Number of emerging AEs during the drug withdrawal period

### Sample size determination

The sample size estimation was based on the results for an analysis of covariance (ANCOVA) comparing changes in the MADRS score from baseline to the last assessment in the 8-week treatment of the study drug or placebo. Reference was made to the results from the phase IIb trial in which the decrease in the MADRS total score in the study drug group was 16.04 and 12.75 in the placebo group. Using a significance level of 5%, a power of 80%, a SD of 8.5, and a dropout rate of 20%, the sample size was estimated to be 133 per group.

### Statistical analysis

Full analysis set for clinical effectiveness analysis and compliance evaluation, and safety analysis set for safety evaluation will be performed in the study. The statistical analysis software will be SAS version 9.4 or above. All statistical tests will be double-sided, and *P*-values less than or equal to 0.05 will be considered statistically significant. The statistical analysis shall be carried out according to the statistical analysis plan. Descriptive statistics for cases will be the percentages (%); descriptive statistics for measurement data will describe the number of cases, mean, standard deviation, median, minimum, and maximum. Change score outcomes will be adjusted for baseline values to prevent regression to the mean.

### Primary efficacy analysis

The treatment effect will be evaluated using ANCOVA with the study drug group, site, depressive episode, and baseline MADRS total score as the explanatory variables. The 95% confidence intervals and *P*-values will be presented. Missing data will be handled using the multiple imputation approach.

### Secondary efficacy analysis

Clinical remission rate as assessed using MADRS and HAMD-17 at the end of the study will be analyzed using ordinal logistic regression as the (proportional) odds ratio of Anyu Peibo versus placebo with a two-sided Wald 95% confidence interval. Treatment, baseline MADRS (total score under beyond or under 30) or HAMD-17 (higher or lower than the median), site, and depressive episode (single episode or relapse) will be included in the model.

The *t*-tests with a two-sided alpha level of 0.05 will be used to analyze the following variables: total MADRS scores, MADRS scores over time, CGI-I scores at different visits, and the changes in total scores from baseline for the HAMD-17, HAMA, CGI-S, DSST, TMT A and B, and SDS. The mean differences in the scores and the 95% confidence intervals for Anyu Peibo compared to placebo will be used as the estimate of the treatment effect.

The estimate of the difference in the proportions of patients withdrawing due to poor efficacy and the proportions of patients using combined medication to treat insomnia (Anyu Peibo versus placebo), 95% confidence intervals, and chi-square *P*-values will be calculated. In the cases of low cell frequencies (< 5), the Fisher exact test will be used instead.

### Trial management, monitoring, and auditing

An independent DSMB is monitoring the quality of the trial and has access to trial outcomes and accumulated safety data, including adherence, SAEs, suspected unexpected serious adverse reactions, and mortality. In addition, the DSMB will review the safety data from a clinical and safety point of view on an ongoing basis.

The sponsor will perform monitoring visits as frequently as necessary. Representatives from the sponsor’s quality assurance department can visit the trial site at any time during the study to conduct an audit of the study in compliance with the regulatory guidance.

## Discussion

Antidepressants are the first-line recommended treatment for MDD, which is a serious psychological disorder with a high disease burden in adults [27, 28]. However, concerns over the long-term balance of benefits and harms arising from the currently marketed antidepressants point to the need for new antidepressants with improved efficacy and safety.

*Piper laetispicum* C. DC. has been used in Chinese traditional medicine as a sedative and analgesic and to treat toothache and snake bites [29]. The n-hexane extracts from *Laetispicum* C. DC. have been tested for their anti-inflammatory activity against cyclooxygenase-1 and 5-lipoxygenase, showing evidence that extracts of these species act as in vitro inhibitors of both enzymes [30–32]. *Piper* alkamides have been reported to possess various properties, including antidepressant effects. Its anxiolytic and antidepressant effects have been explained by its capability to inhibit monoamine oxidase activity and increase the levels of serotonin and noradrenaline in some regions of the mouse brain [33, 34].

Anyu Peibo Capsule includes the extracted *Piper* alkamides (amide alkaloids), and the administration of Anyu Peibo Capsule has been estimated to improve the MDD clinical outcome with a favorable safety profile in phase IIa and IIb trials. This phase III trial aims to demonstrate a beneficial effect of Anyu Peibo Capsule on patients with MDD, while minimizing the side effects, in particular those relating to sexual dysfunction and gastrointestinal discomfort.

### Limitation

The long-term outcomes of Anyu Peibo were not included in this trial. A substantially larger effect will be demonstrated in a superiority trial.

### Trial status

The trial is currently recruiting and enrolling patients according to version 1.2 of the protocol (August 2019). Recruitment began on January 23, 2020, and the approximate date for completion of recruitment will be in December 2020.

## Data Availability

Not applicable.
